# Myeloid Fbxw7 Prevents Pulmonary Fibrosis by Suppressing TGF-β Production

**DOI:** 10.3389/fimmu.2021.760138

**Published:** 2022-01-05

**Authors:** Jia He, Yue Du, Gaopeng Li, Peng Xiao, Xingzheng Sun, Wenjun Song, Lihua Lai, Meng Xia, Jianhua Zhang, Qingqing Wang

**Affiliations:** ^1^ Institute of Immunology, Zhejiang University School of Medicine, Hangzhou, China; ^2^ The Key Laboratory for Immunity and Inflammatory Diseases of Zhejiang Province, Hangzhou, China; ^3^ Department of Medical Laboratory, School of Medicine, Shaoxing University, Shaoxing, China

**Keywords:** IPF – idiopathic pulmonary fibrosis, TGF-β, Fbxw7, macrophage, c-Jun

## Abstract

Idiopathic pulmonary fibrosis (IPF) is a group of chronic interstitial pulmonary diseases characterized by an inexorable decline in lung function with limited treatment options. The abnormal expression of transforming growth factor-β (TGF-β) in profibrotic macrophages is linked to severe pulmonary fibrosis, but the regulation mechanisms of TGF-β expression are incompletely understood. We found that decreased expression of E3 ubiquitin ligase *Fbxw7* in peripheral blood mononuclear cells (PBMCs) was significantly related to the severity of pulmonary fibrosis in IPF patients. Fbxw7 is identified to be a crucial suppressing factor for pulmonary fibrosis development and progression in a mouse model induced by intratracheal bleomycin treatment. Myeloid cell-specific *Fbxw7* deletion increases pulmonary monocyte-macrophages accumulation in lung tissue, and eventually promotes bleomycin-induced collagen deposition and progressive pulmonary fibrosis. Notably, the expression of TGF-β in profibrotic macrophages was significantly upregulated in myeloid cell-specific *Fbxw7* deletion mice after bleomycin treatment. C-Jun has long been regarded as a critical transcription factor of *Tgfb1*, we clarified that Fbxw7 inhibits the expression of TGF-β in profibrotic macrophages by interacting with c-Jun and mediating its K48-linked ubiquitination and degradation. These findings provide insight into the role of Fbxw7 in the regulation of macrophages during the pathogenesis of pulmonary fibrosis.

## Introduction

Idiopathic pulmonary fibrosis (IPF) is a lung disease characterized by fibroblast hyperproliferation and extracellular matrix (ECM) deposition (1), which can cause pulmonary fibrosis and eventually lead to respiratory failure. However, the etiology of IPF remains unclear. Transforming growth factor-β (TGF-β) is one of the best-characterized pro-fibrotic factors (2). In mammals, there are three isoforms of TGF-β (TGF-β1, TGF-β2, and TGF-β3), of which TGF-β1 most closely related to the development of IPF (2). Disturbances of the pulmonary microenvironment induced by TGF-β are critical to promote the recruitment of circulating fibrocytes and bone marrow–derived progenitor cells to the lung (3), and enhance the pro-fibrotic capacities of these cells or induce their differentiation into activated myofibroblasts (4), eventually leading to the typical IPF symptoms like ECM deposition and abnormal collagen accumulation (5). Moreover, TGF-β1 induces macrophage recruitment and stimulates the expression of several pro-inflammatory and profibrogenic cytokines in macrophages, such as TNF-α and IL-1β (5). IPF patients have much higher TGF-β1 mRNA expression in profibrotic macrophages compared to normal volunteers (6, 7). Previous studies have reported that TGF-β1 expression can be induced by cytokines, such as IL-1β, TNF-α (8), IL-13 (9, 10) and IL-24 (11), the increased generation of ROS also induces TGF-β1 production (12). Moreover, the activation of TGF-β1 in fibrotic disorders can be induced by multiple factors, such as matrix metalloproteinases (MMP) 3 and MMP9 (13), thrombospodin-1 (14) and integrins α_V_β_6_ (15). TGF-β1 signaling pathways and their downstream signal transduction mechanisms have been extensively studied, but there are few reports involved in the regulation mechanism of TGF-β1 expression.

Pulmonary macrophages derived from blood monocytes and classified into CD64^+^SiglecF^+^ alveolar macrophages (AMs) and CD11b^+^CD206^+^ interstitial macrophages (IMs) based on the different anatomical locations (16), both of those macrophage subsets derived from blood monocytes have been known to play a crucial role in IPF pathogenesis. Pulmonary macrophages polarized into alternatively activated anti-inflammatory phenotypes to promote tissue repair and fibrosis after lung injury (17). Aberrant immune function of anti-inflammatory macrophages present profibrogenic phenotypes, which induce the dysfunction of wound repair and accumulate in fibrotic lung, further support fibrogenesis of lung by generating reactive oxygen species (ROS) (6), contributing to collagen synthesis and ECM remodeling. Increased accumulation of profibrotic AMs have been detected in lung of IPF patients (6). During lung fibrogenesis, profibrotic macrophages release soluble factors to create and sustain the profibrotic lung microenvironment (18–20), such as TGF-β1, CCL8 (21), IGF-I (22), PDGF (23), and arginase 1 (24). In contrast, loss of profibrotic macrophages promotes the resolution of established pulmonary fibrosis (25) and the reduction of lung collagen deposition (26). Importantly, in rodents with bleomycin-induced lung fibrosis, pulmonary macrophages have been demonstrated to be the predominant source of profibrogenic mediator TGF-β (27). However, the specific mechanism of TGF-β1 secreted by macrophages in the pathogenesis of IPF is remain poorly understood.

F-box and WD repeat domain–containing 7 (Fbxw7) is a member of the F-box protein family, which is a component of the SKP1-cullin-F-box-protein (SCF) ubiquitin ligase complex. Many studies have shown that Fbxw7 is a tumor suppressor by targeting c-Myc, Notch, MCL1 and c-Jun (28). Fbxw7 mutation was detected in squamous cell carcinoma developed on IPF (29). Fbxw7 also participates in the regulation of Parkinson’s disease (30) and circadian rhythm (31). Moreover, Fbxw7 controls neural stem cell differentiation (32) and myelination (33). Fbxw7 has immunoregulatory capacities, it controls effector T cell polyfunctionality and survival in tumor environment by inhibiting Notch activation (34). Our previous studies have shown that Fbxw7 enhances antiviral innate immune response (35) and aggravate intestinal inflammation (36). These observations implicate that Fbxw7 may act as a transducer of tissue microenvironment, regulating the plastic function of macrophages. In this work, we further investigated the function of Fbxw7 in pulmonary macrophages during the pathogenesis of IPF. Our findings demonstrate that *Fbxw7* expression decreased significantly in peripheral blood mononuclear cells (PBMCs) of IPF patients and lung tissues of the mice model of bleomycin-induced fibrosis. *Fbxw7* deletion in myeloid cells increased the release of fibrogenic factors and the recruitment of monocytes into lung tissues. In terms of the mechanism, the *Fbxw7* deletion promoted the TGF-β expression in macrophages through reducing c-Jun ubiquitination, thereby aggravating the severity of pulmonary fibrosis.

## Materials and Methods

### Mice Information

*Fbxw7^fl/fl^* mice (C57BL/6J background) were obtained from Jackson Laboratories. *LysM-Cre* mice C57BL/6J were kindly provided by Dr. Ximei Wu (Zhejiang University School of Medicine, Hangzhou, China). *LysM^+^Fbxw7^fl/fl^* mice were generated by crossing *Fbxw7^f/f^* mice with *LysM-Cre* transgenic mice as previously described (36). All mice were bred at the laboratory animal center of Zhejiang University under specific pathogen-free conditions. In all experiments, age-matched (8-12 week) mice were randomized into the experimental or control groups. All animal experiments were performed according to the protocol approved by the Animal Ethics Committee of Zhejiang University and were in compliance with institutional guidelines.

### Cell Culture and DNA Transfection

Bone marrow cells were isolated by flushing femurs and tibias of 6- to 8-week-old mice with PBS and differentiated into BMDMs in RPMI-1640 medium with 10% (vol/vol) FCS and 10 ng/ml recombinant mouse M-CSF (R&D, 416-ML). To induce M2 macrophages, BMDMs were stimulated by adding the recombinant mouse IL-4 (30 ng/ml, PeproTech, 214-14). Proteasome inhibitors MG132 (M8699), protein synthesis inhibitor CHX (C4859), and JNK inhibitor SP600125 (420119) were from Sigma-Aldrich. TGF-β neutralizing antibody (MAB1835) and mouse IgG1 isotype control (MAB002) were from R&D systems.

Mouse embryonic fibroblast cell line (MEF) was obtained from American Type Culture Collection (ATCC) and maintained in Dulbecco’s modified Eagle medium (DMEM) with 10% fetal bovine serum (FBS; BI). Murine macrophage-like RAW264.7 cells were cultured in RPMI 1640 medium with 10% fetal bovine serum (FBS; BI), 100 U/ml penicillin and 100 mg/ml streptomycin in an atmosphere of humidified 5% CO_2_ at 37°C.

Recombinant vectors encoding mouse c-Jun (NM_010591) were cloned into pcDNA3.1-c-Myc eukaryotic expression vector. The RAW264.7 cells (3 × 10^5^ cells/well, in 24-well plate) were incubated with NATE™ (*In vivo*gen, lyec-nate) for 30 min to enhance the transfection efficiency and transient expression of target plasmid. Then RAW264.7 cells were transfected with c-Jun plasmid (Roche, FuGENE HD Reagent) and treated with IL-4 for the indicated time.

### Co-Culture Experiments

MEF cells (2×10^5^) were cultured in the lower chamber and macrophages (5×10^5^) were added to the upper chamber of a 12-well transwell apparatus (0.4 mm pore size, Costar, Cambridge, MA). The MEF were subjected to the further analysis at 48 h after co-culturing.

### Induction of Pulmonary Fibrosis

An experimental pulmonary fibrosis model was established using the direct endotracheal injection of bleomycin (37). Briefly, 6- to 8-week-old C57BL/6 mice were anesthetized with pentobarbital by intraperitoneal injections. Neck area of mice was sterilized using povidone-iodine swabs after depilation of hairs, and 1 cm midline incision was made with sterile scissors. Intratracheal administration of 3.5 mg/kg bleomycin hydrochloride (Hisun-Pfizer Pharmaceuticals, Taizhou, China) in sterile PBS, an equal volume of sterile PBS for controls by using 1 ml syringe fitted with a 26 G needle (carefully insert the needle into the visualized trachea and rapidly inject the bleomycin solution during a single inspiration). The incision was closed with surgical sutures after withdrawing the needle. The mice were placed on warmer pads and monitored until fully awake. Mouse lung tissues were sacrificed at 14 days and 21 days post-bleomycin injection for subsequent experiments.

LPS induced pulmonary injury model (38, 39) were established using the direct endotracheal injection of 5 mg/kg LPS (Sigma-Aldrich, St. Louis, Missouri, USA). Mouse lung tissues were sacrificed at 10 days post-LPS injection for subsequent experiments.

### Flow Cytometry

Cells were stained with fluorochrome-labeled mAbs and analyzed with an LSRII flow cytometer (BD). Flow cytometric analysis was performed using FlowJo software. The following fluorochrome-labeled mAbs were purchased from Biolegend, Inc. and used according to the manufacturers’ protocols: anti-CD11b-FITC (101205, RRID: AB_312788), anti-CD206-APC (141707, RRID: AB_10896057), anti-Ly6C-APC-Cy7 (128026, RRID: AB_10640120), anti-Ly6G-PE (127608, RRID: AB_1186099), anti-F4/80-Brilliant Violet 421 (123132, RRID: AB_11203717), anti-CD64-PE (139303, RRID: AB_10613467), and anti-Siglec-F-APC (155507, RRID: AB_2750236).

### Immunoprecipitation and Immunoblot Analysis

Immunoprecipitation, SDS-PAGE and immunoblot analysis were performed according to a standard protocol as described previously (35). The following antibodies were used according to the manufacturers’ protocols: anti-Fbxw7 (Abcam, ab12292, RRID: AB_442966), anti-Lys48-specific linked polyubiquitin (MilliporeSigma, 05-1307, RRID: AB_1587578), anti-c-Jun (CST, #9165S, RRID: AB_2130165), anti-p65 (CST, #8242, RRID: AB_10859369), anti-p-p65 (CST, #3036, RRID: AB_331281), Collagen-I(Abcam, ab34710, RRID: AB_731684), Collagen-III (Abcam, ab7778, RRID: AB_306066), EGR-1 (CST, #4154S, RRID: AB_2097035). The PVDF membranes were incubated with corresponding primary antibodies followed by horseradish peroxidase-linked secondary antibodies. The images were developed and captured using Chemiluminescence imaging system (ChemiScope 3000 Mini, Clinx Science Instruments Co., Ltd). Relative quantities of target protein were determined comparing to β-actin expression using densitometric analysis (ImageJ 1.52v). The standard deviation was calculated for biological duplicates.

### Lung Histology

Lungs from bleomycin-treated or control mice were dissected, fixed in 10% phosphate-buffered formalin, and embedded into paraffin. Tissue sections were stained with H&E for morphological analysis and with Masson’s trichrome for the detection of collagen fibers according to the manufacturers’ instructions (Nanjing Jiancheng, Nanjing, China). The Szapiel’s score was used to evaluate the severity of fibrosis using the following criteria (40): none (0), no evidence of fibrosis; mild (1), <20% of the lung affected; moderate (2), 20%–50% affected; severe (3), >50% affected.

### Hydroxyproline Assay

To assess collagen synthesis in the lung, pulmonic hydroxyproline levels were determined using a hydroxyproline assay kit obtained from Jiancheng Bioengineering Institute (Nanjing, China), according to manufacturer’s instructions. Hydroxyproline content was expressed as μg/mg lung wet weight.

### Quantitative RT-PCR

Total RNA was extracted from tissues or cells using RNAiso Plus (TakaRa), and then reversely transcribed to cDNA using the PrimeScript RT reagent kit (TaKaRa) with random primers according to the manufacturer’s instructions. Quantitative PCR was performed using KAPA SYBR Green (SYBR Green Fast qPCR Master Mix) on CFX96 Touch™ Real-Time PCR Detection System (BioRad). Expression data were normalized to the mRNA levels of the β-actin gene and calculated using the 2^−ΔΔCt^ method (41). The sequences for primers were listed in **Supplementary Table 1**.

### ELISA

The levels of mouse TGF-β in bronchoalveolar lavage fluid (BALF) or culture supernatants were measured with ELISA kits (Invitrogen, 88-8350-88) according to the manufacturer’s instructions.

### Statistical Analysis

Statistical analysis was performed with GraphPad Prism 7.0. All data are shown as mean ± SD. The comparisons of two experimental groups were determined using an unpaired, 2-tailed student’s *t*-test. Multiple comparisons were assessed by one-way ANOVA with Tukey’s multiple comparisons test. *P-*values less than 0.05 were considered significant.

## Results

### Decreased Expression of *Fbxw7* Is Related to IPF

**Figure 1:**
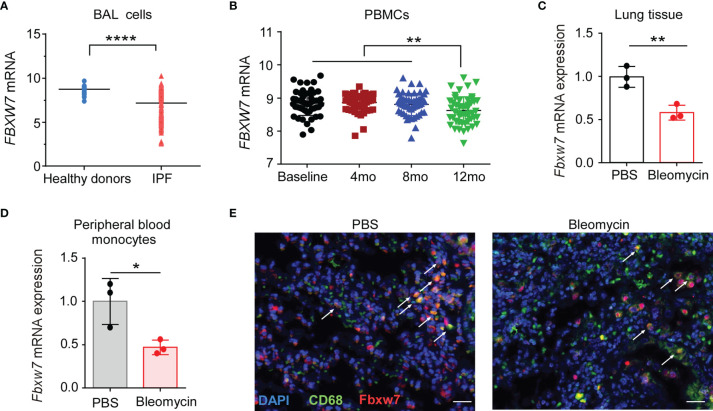
Decreased Fbxw7 expression is related to pulmonary fibrosis. **(A)** Fbxw7 mRNA expression in bronchoalveolar lavage (BAL) cells from healthy donors (n=48) and from IPF patients (n=176), the data were collected from GEO database (GSE70867), P value was obtained using two-tailed Student’s *t* test, ****P < 0.0001. **(B)** *Fbxw7* mRNA expression in PBMCs from baseline (n=74), 4 months (4 mo, n=74), 8 months (8 mo, n=68) and 12 months (12 mo, n=60) IPF patients, the data were collected from the GEO database (GSE132607). Statistical significance was assessed by one-way ANOVA with Tukey’s multiple comparisons test, **P < 0.01. qRT-PCR analysis of lung tissues **(C)** and peripheral blood monocytes **(D)** obtained from mice after 21 days of bleomycin-induced lung injury and healthy controls. Data are expressed as mean ± SD of biological duplicates (n=3) and are representative of three independent experiments. P value was obtained using two-tailed Student’s *t* test, *P < 0.05, **P < 0.01. **(E)** Immunofluorescence staining for FBXW7 (red), CD68 (green), and DAPI for nuclei (blue) in lung tissues obtained from mice after 21 days of bleomycin-induced lung injury and healthy control mice. Scale bars: 20 μm. Data shown are the representative of three independent experiments.

We first investigated whether Fbxw7 was related to the development of IPF, the expression level of *Fbxw7* from IPF patients was analyzed in the data from Gene Expression Omnibus (GEO) database (GSE70867) (42). The result showed that *Fbxw7* mRNA expression in bronchoalveolar lavage (BAL) cells of IPF patients was significantly lower than that in healthy controls (**Figure 1A**). In another database of microarray expression profiling of PBMCs from IPF patients in the training cohort (GSE132607) (43), *Fbxw7* mRNA expression level in 12-months IPF patients was significantly lower than that in baseline, 4-months, and 8-months IPF patients (**Figure 1B**). We then used the bleomycin-induced mouse model of pulmonary fibrosis to observe the expression of *Fbxw7* in lung tissues (**Figure 1C**) and peripheral blood monocytes (**Figure 1D**). qRT-PCR results showed that the expression level of *Fbxw7* was significantly reduced after 21 days of intratracheal bleomycin administration compared to the PBS controls. Moreover, the expression of *Fbxw7* in the macrophages in mice pulmonary fibrosis tissue was markedly decreased compared with those in healthy control subjects detected by immunofluorescence staining (**Figure 1E**). These results suggest that the decreased expression of Fbxw7 is associated with the formation of pulmonary fibrosis.

### Myeloid Deficiency of *Fbxw7* Aggravates Bleomycin-Induced Collagen Deposition in Lung Tissue

Augmented collagen deposition within the interstitium (44, 45) and the remodeling of ECM (46) are recognized as one of the primordial pathophysiological events of IPF. To investigate the role of Fbxw7 in macrophages in the pathogenesis of IPF, we generated LysM-Cre^+^Fbxw7^fl/fl^ (*LysM^+^Fbxw7^fl/fl^*) mice (**Supplementary Figure 1A**) and confirmed that myeloid specific deletion of *Fbxw7* does not affect the differentiation and development of myeloid cells (**Supplementary Figures 1B-F**).

**Figure 2:**
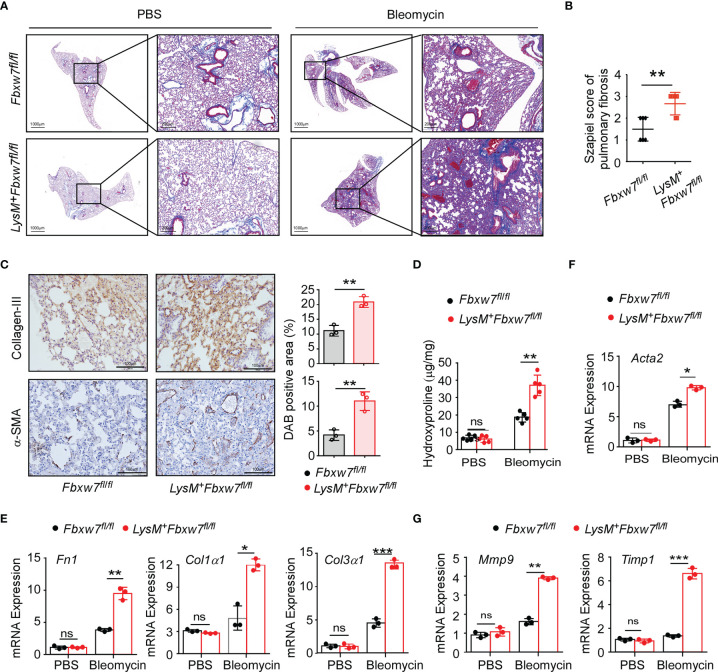
*LysM^+^Fbxw7^fl/fl^* mice show aggravated pulmonary fibrosis. *Fbxw7^fl/fl^* and *LysM^+^Fbxw7^fl/fl^* mice were administered bleomycin by direct endotracheal injection to induce pulmonary fibrosis. Three weeks later, lung tissues were isolated for Masson staining and RT-PCR analysis. **(A)** Masson staining of collagen fiber, scale bars 1000 μm (whole pulmonary section), and 200 μm (detail) and sapiel score of pulmonary tissue **(B)** obtained from *Fbxw7^fl/fl^* and *LysM^+^Fbxw7^fl/fl^* mice. **(C)** Representative collagen III and α-SMA immunohistochemical staining of lung tissues obtained from *Fbxw7^fl/fl^* and *LysM^+^Fbxw7^fl/fl^* mice after 21 days of bleomycin-induced lung injury, scale bars 100 μm. Comparison of DAB staining intensity (Image J) among the groups is shown. Quantification of hydroxyproline contents **(D)** and relative mRNA expression of collagen genes **(E)**, Acta2 gene (α-SMA) **(F)**, and matrix metalloproteinases **(G)** in the lungs from *Fbxw7^fl/fl^* and *LysM^+^Fbxw7^fl/fl^* mice after bleomycin treatment for 21 days. Data are expressed as mean ± SD of biological duplicates (n≥3) and are the representative of three independent experiments. P values were obtained using two-tailed Student’s *t* test. *P < 0.05, **P < 0.01, ***P < 0.001, ns, not significant.

*LysM^+^Fbxw7^fl/fl^* mice and their *Fbxw7^fl/fl^* littermates were subjected to establish experimental pulmonary fibrosis model. Masson staining of lung tissues showed that collagen deposition was significantly increased in *LysM^+^Fbxw7^fl/fl^* mice 14 days after bleomycin administration, compared with *Fbxw7^fl/fl^ mic*e (**Supplemental Figure 2A**), and further increased after 21 days (**Figure 2A**). Szapiel scores also showed that Fbxw7 knockout exacerbated the degree of fibrosis (**Supplemental Figure 2B** and **Figure 2B**). The expression of collagen III and α-SMA observed by immune-histochemical staining was also significantly higher in the fibrotic lung tissue of *LysM^+^Fbxw7^fl/fl^* mice compared with *Fbxw7^fl/fl^* littermates (**Figure 2C**). Furthermore, *LysM^+^Fbxw7^fl/fl^* mice had significantly higher levels of hydroxyproline in lung tissues compared with *Fbxw7^fl/fl^* mice (**Figure 2D**).

Moreover, we examined the mRNA expression levels of collagen-related genes in the lung tissues after bleomycin administration. Fibronectin 1 (Fn1), a pro-fibrotic marker, is an important member of the fibronectin family (47). Col1α1 and Col3α1 is component of the ECM in IPF. qRT-PCR results showed that the expression of *Fn1* and *Col3α1* increased significantly in *LysM^+^Fbxw7^fl/fl^* mice (**Supplemental Figure 2C** and **Figure 2E**). Furthermore, as a surface molecular marker of myofibroblasts, the mRNA expression of α-smooth muscle actin (*α-SMA* or *Acta2*) was also significantly increased in *LysM^+^Fbxw7^fl/fl^* mice (**Supplemental Figure 2D** and **Figure 2F**). Matrix metalloproteinases (MMPs) and their specific inhibitors (tissue inhibitors of metalloproteinases, TIMPs) participate in the formation and degradation of ECM, and the imbalance between MMPs and TIMPs plays a pivotal role in pulmonary fibrosis pathogenesis. qRT-PCR results demonstrated that *Fbxw7* deletion significantly increased the expression of *Mmp9* and *Timp1* (**Figure 2G**). These results suggest that the myeloid cell specific *Fbxw7* knockout mice have increased collagen deposition in their lung tissue that may aggravate the degree of fibrosis induced by bleomycin.

### Myeloid Deficiency of *Fbxw7* Aggravates Bleomycin-Induced Lung Injury and Monocytes Recruitment

**Figure 3:**
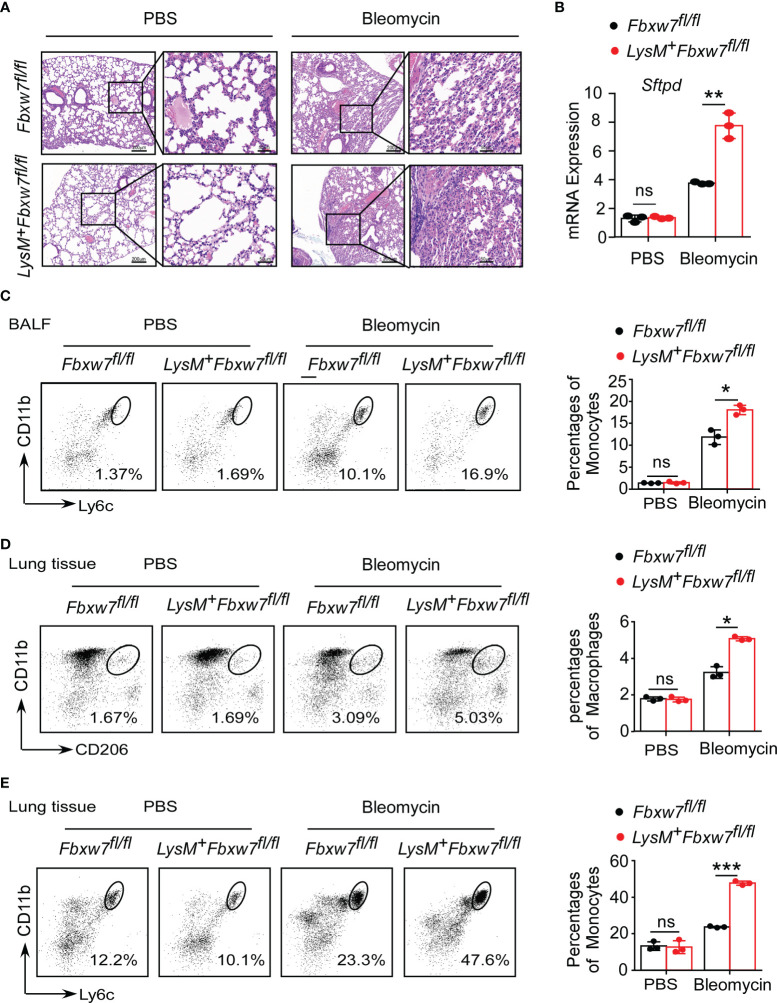
*Fbxw7* deficiency increases recruitment of monocytes and macrophages after bleomycin-induced lung injury. *Fbxw7^fl/fl^* and *LysM^+^Fbxw7^fl/fl^* mice were administered PBS or bleomycin for 21 days to induce pulmonary fibrosis. **(A)** H&E staining of lung sections, scale bars 200 μm (whole pulmonary section), and 50 μm (detail). **(B)** qRT-PCR analysis of relative expression of *Sftpd* in lung tissue. Flow cytometry and statistical analysis of the percentage of CD11b^+^Ly6C^+^ monocytes in bronchoalveolar lavage fluid (BALF) **(C)**, CD11b^+^CD206^+^ IMs **(D)** and CD11b^+^Ly6C^+^ monocytes **(E)** in lung tissues, data are expressed as mean ± SD of biological duplicates (n=3) and are the representative of three independent experiments. P values were obtained using two-tailed Student’s *t* test. *P < 0.05, **P < 0.01, ***P < 0.001, ns, not significant.

Tissue damage and inflammation are important triggers for pulmonary fibrosis (48). To determine whether *Fbxw7* deletion in myeloid cells aggravates lung injury and inflammation, we evaluated the morphological characteristics in mouse lung tissues. The H & E staining results showed that the lung tissues of *Lysm^+^Fbxw7^fl/fl^* mice were more severely damaged 14 days (**Supplemental Figure 3A**) and 21 days (**Figure 3A**) after bleomycin administration. Lung tissues of *LysM^+^Fbxw7^fl/fl^* mice appeared to be denser, congestion and edema, as well as loss of alveolar structures compared with *Fbxw7^fl/fl^* mice. Moreover, we examined the mRNA expression of surfactant protein-D (*Sftpd*) and qRT-PCR results showed that myeloid deficiency of *Fbxw7* resulted in higher levels of *Sftpd* in fibrotic lung tissues (**Supplemental Figure 3B** and **Figure 3B**).

Furthermore, *LysM^+^Fbxw7^fl/fl^* mice had higher degree of inflammatory cell infiltration in the alveolar space observed by H & E staining (**Figure 3A**). Flow cytometric analysis was used to investigate the changes in mononuclear phagocyte subpopulations in bronchoalveolar lavage fluid (BALF) and lung tissue during pulmonary fibrosis development. After 21 days of bleomycin-induced pulmonary fibrosis in mice, *Fbxw7* knockout did not increase CD64^+^SiglecF^+^ AMs accumulation (**Supplemental Figure 3C**), but significantly increased CD11b^+^Ly6C^+^ monocytes recruitment (**Figure 3C**) in BALF. Moreover, a single-cell suspension was prepared by digesting lung tissues and analyzed by flow cytometry. Compared with the PBS group, the percentages of CD11b^+^CD206^+^ IMs (**Figure 3D**) and CD11b^+^Ly6C^+^ monocytes (**Figure 3E**) in lung tissues of mice treated with bleomycin were increased. Importantly, *LysM^+^Fbxw7^fl/fl^* mice showed an obviously increased accumulation of IM and monocyte cell populations compared with *Fbxw7^fl/fl^* littermates (**Figures 3D, E**). These results suggest that the deletion of *Fbxw7* exacerbates injury-induced lung fibrosis by promoting the recruitment and accumulation of mononuclear phagocytes.

### *Fbxw7* Deficiency Upregulates TGF-β Expression in Macrophages

**Figure 4:**
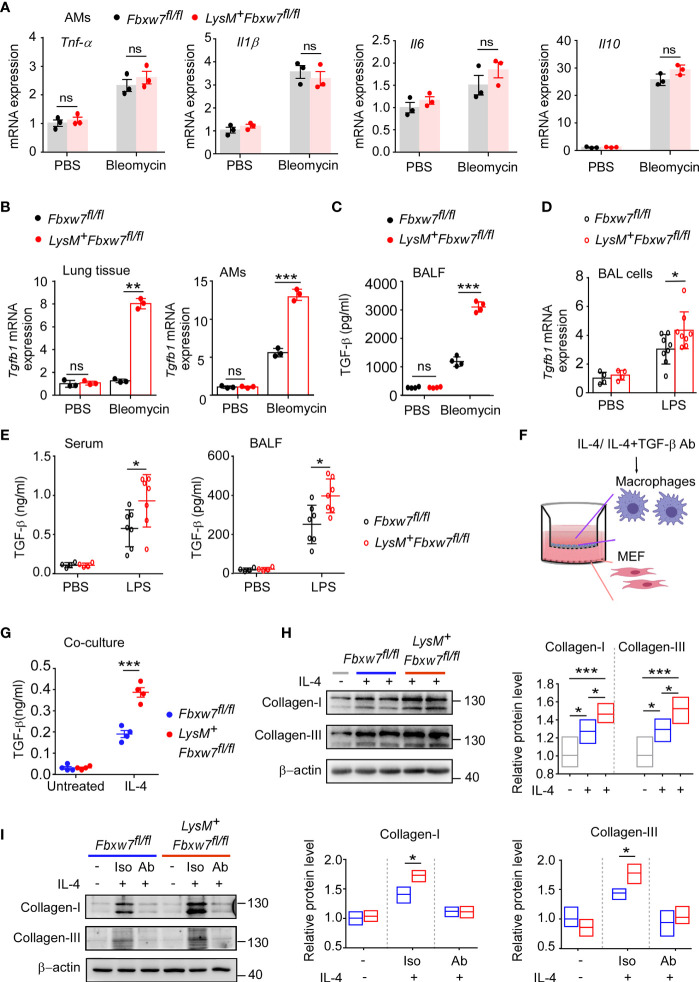
*Fbxw7* deletion upregulates TGF-β expression in lung fibrosis model induced by bleomycin. qRT-PCR analysis of *Tnf-α*, *Il1β*, *Il6*, and *Il10* relative mRNA expression of AMs **(A)** after *Fbxw7^fl/fl^* and *LysM^+^Fbxw7^fl/fl^* mice were administered PBS or bleomycin for 21 days to induce pulmonary fibrosis. After a 21d period of bleomycin-induced lung injure, qRT-PCR analysis of *Tgfb1* relative mRNA expression in lung tissue and AMs **(B)**, TGF-β content in BALF was detected by ELISA **(C)**. *Tgfb1* relative mRNA expression in BAL cells was analyzed by qRT-PCR **(D)**, TGF-β content in serum and BALF was detected by ELISA **(E)** after 10 days of LPS-induced lung injury. Macrophages from *Fbxw7^fl/fl^* and *LysM^+^Fbxw7^fl/fl^* mice were stimulated with IL-4 (30 ng/ml) and treated with or without TGF-β neutralizing antibody for 12 h, then co-cultured with MEF for another 48 h. **(F)** The schematics of the approach. **(G)** TGF-β in the media of the macrophage co-culture system. Data are expressed as mean ± SD of biological duplicates (n≥3) and are the representative of three independent experiments. P values were obtained using two-tailed Student’s *t* test, *P < 0.05, **P < 0.01, ***P < 0.001, ns, not significant. **(H)** Representative images of western blot analysis of collagen-I and collagen-III in co-cultured MEF after IL-4 treatment. Densitometric fold change of target proteins based on IL-4 untreated group was calculated. **(I)** Representative images of western blot analysis of collagen-I and collagen-III in co-cultured MEF after IL-4 with TGF-β neutralizing antibody (Ab) treatment (3 μg/ml). Iso: Isotype. Relative protein levels were presented by densitometric fold change quantified by ImageJ based on *Fbxw7^fl/f^* untreated group, the floating bars indicate the min, mean and max value of biological duplicates (n=3), which are the representative of three independent experiments. P values were obtained using One-way ANOVA with Tukey’s Multiple Comparison test **(H)** or two-tailed Student’s *t* test **(I)**. *P < 0.05, **P < 0.01, ***P < 0.001.

Macrophages are the major source of multiple profibrotic mediators in pulmonary fibrosis (49). We next investigated whether myeloid *Fbxw7* deletion altered the cytokine profile in fibrotic lung tissues. The qRT-PCR results showed that the mRNA expressions of *Tnf-α*, and *Il1β* in lung tissues of *LysM^+^Fbxw7^fl/fl^* mice was significantly higher than that of *Fbxw7^fl/fl^* mice 14 days after bleomycin administration, but the expression of *Il6* and *Il10* was not significantly different (**Supplemental Figure 4A**). In contrast, *LysM^+^Fbxw7^fl/fl^* mice had significantly higher levels of *Il6* and *Il1β*, but not *Tnf-α* and *Il10*, compared with *Fbxw7^fl/fl^* mice 21 days after administration of bleomycin (**Supplemental Figure 4B**). Furthermore, we evaluated cytokines expression in alveolar macrophages and found that the mRNA expressions of *Il1β, Tnf-α, Il6* and *Il10* were significantly increased after bleomycin administration, however, there were no significant differences in the expression level between *LysM^+^Fbxw7^fl/fl^* mice and *Fbxw7^fl/fl^* littermates (**Figure 4A**). Interestingly, mRNA expression of *Tgfb1*, the macrophage-derived fibrogenic cytokine, was significantly increased in lung tissues and alveolar macrophages in *LysM^+^Fbxw7^fl/fl^* mice compared to *Fbxw7^fl/fl^* littermates (**Figure 4B**). ELISA further confirmed that the concentration of TGF-β protein in BALF from *LysM^+^Fbxw7^fl/fl^* mice was significantly higher than that from *Fbxw7^fl/fl^* littermates (**Figure 4C**). In lung injury model established by LPS, more severe lung injury, fibrosis associated collagen deposition as well as higher expression of *Tgfb1* were found in lung tissue and BAL cells from *LysM^+^Fbxw7^fl/fl^* mice compared to *Fbxw7^fl/fl^* littermates (**Supplemental Figures 4C, D** and **Figure 4D**). The significantly elevated concentration of TGF-β protein in BALF and serum from *LysM^+^Fbxw7^fl/fl^* mice was observed by ELISA compared with that from *Fbxw7^fl/fl^* littermates (**Figure 4E**). These data suggest that deficiency of *Fbxw7* in macrophages exacerbates pulmonary fibrosis, which may be related to the up-regulation of TGF-β expression.

IL-4, as a Th2 cytokine, was reported to induce TGF-β expression in profibrotic macrophage to activate fibroblasts and promotes extensive tissue fibrosis (50, 51). qRT-PCR confirmed that the expression of *Il4* was abundant in fibrotic lung tissues, but the level of *Il4* was similar between *LysM^+^Fbxw7^fl/fl^* mice and their *Fbxw7^fl/fl^* littermates (**Supplemental Figure 4E**). Next, we performed a Transwell co-culture assay to evaluate the profibrogenic capability of macrophages from *LysM^+^Fbxw7^fl/fl^* mice and *Fbxw7^fl/fl^* littermates after IL-4 treatment *in vitro* (**Figure 4F**), the results consistently showed that myeloid *Fbxw7* deficiency resulted in higher secretion levels of TGF-β in co-cultured macrophages after IL-4 treatment (**Figure 4G**), *Fbxw7* deficient macrophages treated with IL-4 mediates a higher activation of co-cultured fibroblasts by the examination of the expression levels of collagen-I and collagen-III through western blot (**Figure 4H**). To further confirm that the enhanced profibrogenic capability of macrophages from *LysM^+^Fbxw7 ^fl/fl^* mice is TGF-β-dependent, transwell co-culture assay was performed after IL-4 treatment with or without TGF-β neutralizing antibody. Neutralization of TGF-β abrogated the difference of profibrogenic ability between *LysM^+^Fbxw7^fl/fl^* macrophages and *Fbxw7^fl/fl^* macrophages (**Figure 4I**). Collectively, these data further support that *Fbxw7* deletion in myeloid cells can exacerbate bleomycin-induced production of TGF-β and promote the development of pulmonary fibrosis.

### Fbxw7 Regulates TGF-β Expression by Mediating the Ubiquitination and Degradation of c-Jun

**Figure 5:**
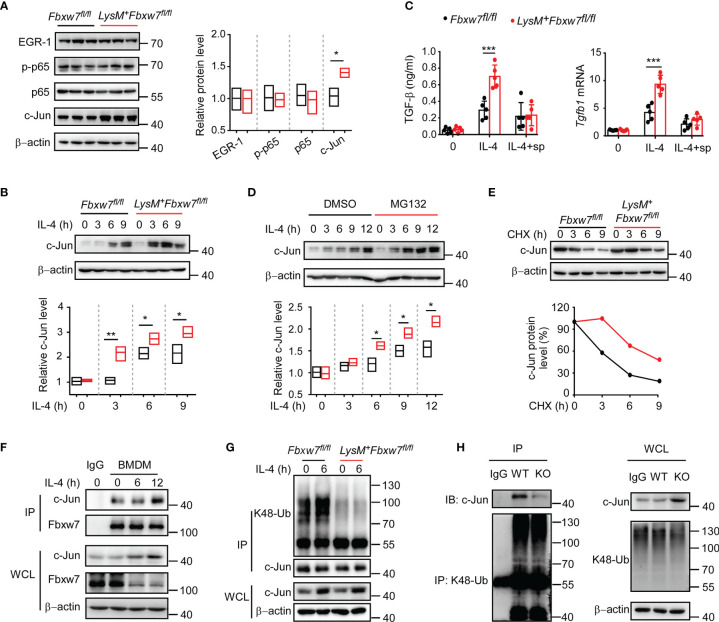
Fbxw7 regulates TGF-β expression *via* mediating the ubiquitination of c-Jun. **(A)** Immunoblot analysis of phosphorylated and/or total proteins in lysates of lung tissues obtained from bleomycin-treated *Fbxw7^fl/fl^* and *LysM^+^Fbxw7^fl/fl^* mice. Densitometric fold change of target proteins based on *Fbxw7^fl/fl^* group was calculated. **(B)** Immunoblot analysis of c-Jun protein levels in lysates of IL-4 (30 ng/ml) stimulated BMDMs from *Fbxw7^fl/fl^* and *LysM^+^Fbxw7^fl/fl^* mice. Densitometric fold change for the relative c-Jun level based on *Fbxw7^fl/fl^* untreated group was calculated. **(C)** *Fbxw7^fl/fl^* and *LysM^+^ Fbxw7^fl/fl^* BMDMs stimulated with IL-4 and treated or not with SP600125 (15 μM), qRT-PCR analysis for *Tgfb1* relative mRNA expression and ELISA for TGF-β protein expression. Data are expressed as mean ± SD of biological duplicates (n=5) and are the representative of three independent experiments. P values were obtained using two-tailed Student’s *t* test, ***P < 0.001. **(D)** Immunoblot analysis of c-Jun expression in BMDMs treated or not with MG132 (20 μM) for 8 hours and stimulated with IL-4 for the indicated durations. Densitometric fold change of c-Jun based on IL-4, MG132 untreated group was calculated. **(E)** Immunoblot analysis and relative protein levels of c-Jun in lysates of *Fbxw7^fl/fl^* and *LysM^+^Fbxw7^fl/fl^* BMDMs treated with CHX (40 μg/ml) for indicated hours after stimulation with IL-4 for 6 h. **(F)** Coimmunoprecipitation and immunoblot of BMDMs stimulated with IL-4 for indicated hours. **(G)** Immunoblot analysis of the K48 ubiquitination of c-Jun in *Fbxw7^fl/fl^* and *LysM^+^Fbxw7^fl/fl^* BMDMs stimulated with IL-4 for 6 h. **(H)** Peritoneal macrophages from *LysM^+^Fbxw7^fl/fl^* (WT) and *Fbxw7^fl/fl^* (KO) mice were stimulated with IL-4 for 6 h, coimmunoprecipitation was performed with K48-Ub to pull-down ubiquitylated proteins, followed by IB with c-Jun. Relative protein levels were presented by densitometric fold change quantified by ImageJ, the floating bars indicate the min, mean and max value of biological duplicates and are the representative of three independent experiments. P values were obtained using two-tailed Student’s *t* test. *P < 0.05, **P < 0.01, ***P < 0.001.

Transcription factors including early growth response protein 1 (EGR-1) (52, 53) and NF-κB (54) have been reported to be involved in the pathogenesis of fibrosis *via* enhancing TGF-β dependent signaling. To further elucidate the mechanisms how Fbxw7 regulates TGF-β production in profibrotic macrophages, we investigated the effect of *Fbxw7* deletion on the protein levels of EGR-1 and NF-κB by western blot. The protein levels of EGR1 and NF-κB in alveolar macrophages after bleomycin stimulation were not significantly changed between *Fbxw7^fl/fl^* and *LysM^+^Fbxw7^fl/fl^* mice, but the level of c-Jun was significantly higher in *LysM^+^Fbxw7^fl/fl^* mice than that in *Fbxw7^fl/fl^* mice (**Figure 5A**). Subsequently, we found significantly higher protein expression of c-Jun in IL-4-stimulated BMDMs after *Fbxw7* deletion (**Figure 5B**). We then overexpressed c-Jun in RAW264.7 cells (**Supplemental Figure 5A**) and analyzed the expression of TGF-β by qRT-PCR and ELISA, the results showed that the expression level of TGF-β was significantly increased after c-Jun overexpression (**Supplemental Figure 5B**), indicating that c-Jun expression is sufficient for TGF-β in macrophages. SP600125 is a specific JNK inhibitor that competes with ATP to inhibit the phosphorylation of c-Jun (55). qRT-PCR analysis and ELISA results showed that the mRNA and protein expression of TGF-β increased significantly after the deletion of *Fbxw7* (**Figures 4C, D**), but this effect was abrogated after inhibition of c-Jun phosphorylation by SP600125 (**Figure 5C**). The MEF cells, co-cultured with peritoneal macrophage from *LysM^+^Fbxw7^fl/fl^* mice or *Fbxw7^fl/fl^* littermates after inhibiting the phosphorylation of c-Jun, showed similar protein levels of collagen-I and collagen-III, which were detected by western blot (**Supplemental Figures 5C, D**). Taken together, *Fbxw7* deletion leads to the upregulation of TGF-β expression and severity of pulmonary fibrosis through the regulation of c-Jun.

It has been demonstrated that the transcription factor c-Jun can be degraded by the ubiquitin-proteasome system as a substrate for Fbxw7 (56). As expected, western blot analysis showed that the degradation of c-Jun protein could be inhibited by protease inhibitor MG132 (**Figure 5D**). A cycloheximide chase assay showed that *Fbxw7* deletion extended the half-life of endogenous c-Jun protein in BMDMs (**Figure 5E**). Immunoprecipitation assay results showed that Fbxw7 can physically bind to c-Jun protein (**Figure 5F**) and induce K48-linked polyubiquitination of c-Jun (**Figure 5G**). Moreover, immunoprecipitation assay was performed by using K48-Ub to pull-down c-Jun and the peritoneal macrophages from *LysM^+^Fbxw7^f/f^* mice showed weaker ability of mediating c-Jun ubiquitination and proteasomal degradation compared with cells from *Fbxw7^f/f^* littermates (**Figure 5H**). This result further revealed that the myeloid *Fbxw7* deficiency leads to a significantly accumulation of c-Jun by abolishing its K48 ubiquitination. Collectively, these data confirmed that Fbxw7 promotes TGF-β expression by inducing K48-linked polyubiquitination and proteasome degradation of c-Jun in macrophages.

## Discussion

The E3 ubiquitin ligase Fbxw7 has been well characterized as a critical tumor suppressor that can mediate the degradation of various oncoproteins by the ubiquitin-proteasome system (57). Our studies and others have presented compelling evidence that Fbxw7 plays a crucial role in inflammation and antivirus immune response through regulating the function of macrophages (35, 36, 58). In this study, we demonstrated that the deletion of *Fbxw7* in the myeloid lineages aggravates bleomycin-induced lung injury, collagen deposition, and monocyte recruitment, and eventually leads to the exacerbation of pulmonary fibrosis in mice. We revealed its underlying mechanism that *Fbxw7* deletion will upregulate TGF-β expression by inhibiting the degradation of c-Jun in macrophages. These findings will be beneficial for understanding the role of innate immune response in the pathogenesis of IPF.

Macrophages are the most abundant immune cell population in the lung environment and are involved in tissue repair and lung fibrosis progression, it has also been recognized to play a significant role in driving the fibrogenesis and matrix remodeling. Given the high plasticity of lung macrophages, the relative roles of tissue-resident alveolar macrophages, interstitial macrophages and monocyte-derived infiltrating macrophages in pulmonary fibrosis is complicated (59). High numbers of both interstitial and alveolar macrophages were detected in pulmonary fibrosis (24), the depletion of macrophages, including alveolar and infiltrating macrophage, reduces fibrogenesis (25, 60). Experimental fibrosis leads to the recruitment of bone marrow-derived AMs that are more profibrogenic than their disrupted resident counterparts (61). Selectively targeting alveolar macrophage differentiation without affecting global monocytes or tissue-resident alveolar macrophage may ameliorate fibrosis depletion, while the depletion of tissue-resident AMs cannot alter fibrosis severity (61). In addition, IMs are also thought to arise from monocytes, and highly expression of monocyte-related genes, such as *Cd14*, *Cd163*, and *Csfr1* (62), activated IMs have been reported to induce myofibroblastic activation and ECM production (24). Furthermore, those more profibrogenic macrophages which play a vital role in airway remodeling in pulmonary fibrosis biases alternatively activated macrophages, so we used IL-4-stimulated BMDMs for *in vitro* experiments to mimic profibrogenic macrophages *in vivo*. Our data showed that more monocytes are recruited into the lung tissues and BALF of *Fbxw7*-KO mice, these monocytes further differentiate into profibrogenic macrophages and then participate in the development of pulmonary fibrosis.

The abundant expression of *Fbxw7* mRNA and protein can be detected in most human and mice tissues. The decreased level of *Fbxw7* mRNA in IPF may be regulated by many factors, including DNA or RNA silencing. The expression of microRNA-155 (miR-155) has been reported to be correlated to the damage degree of IPF (63), and miR-155 reduces the levels of *Fbxw7* mRNA (64). The *Fbxw7* promoter can be regulated through epigenetic mechanism during lung fibrosis, including histone modifications. Aberrant elevation of DNA methyltransferase 1 (Dnmt1) can mediate the hypermethylation of *Fbxw7* promoter and result in decreased gene level of *Fbxw7* (65), subsequently contributes significantly to the development of pulmonary fibrosis (66). Moreover, the expression of enhancer of zeste homolog 2 (Ezh2) is increased in IPF patients, increased Ezh2 recruitment and hypermethylated H3K27 at the promoter of fibrotic related genes have been demonstrated, such as *Cox-2* (67) and *Cxcl10* (68). We speculate the function of Ezh2 in inducing the increase of H3K27me3 at *Fbxw7* promotor and silencing of *Fbxw7* gene (34) might also be involved in regulating pulmonary fibrosis. IL-4 has been shown to induce TGF-β expression in profibrotic macrophage to activate fibroblasts and promotes extensive tissue fibrosis (50). In our study, we found IL-4 was highly expressed in lung fibrosis tissue of bleomycin-induced mice, and the expression of Fbxw7 was significantly decreased in bleomycin-induced mice lung issue and in BMDMs after IL-4 stimulation as shown in **Figures 1**, **S4B** and **5G**. These findings suggest that IL-4 in the profibrotic microenvironment might downregulate Fbxw7 expression and block c-Jun degradation.

TGF-β can be produced by a wide variety of cell types, including lung macrophages, alveolar epithelial cells, endothelial cells, fibroblasts and myofibroblasts. However, lung macrophages are revealed to be the main source of TGF-β (6, 69). In bleomycin induced pulmonary fibrosis, macrophages induce fibroblast differentiation and proliferation by producing TGF-β, thereby exacerbating pulmonary fibrosis (70, 71). The widespread accumulation of collagen in lungs, which was observed in WT mice, disappeared in the myeloid *TGF-β* deficient mice, which showed similar lung architecture to saline controls (6). Several factors have previously been implicated in the transcriptional induction of *Tgfb1*, bacterial induction of *Tgfb1* mRNA expression in human intestinal epithelial cells is butyrate and SP1-dependent (72). P38/MAPK signal, MEK-1 and JNK are involved in *Tgfb1* transcription (73). The higher mRNA expression of *Tgfb1* was detected in *Egr-1* overexpressed mesangial cell compared with control cells (74). Moreover, p65 and c-Jun were recruited to the promoter of *Tgfb1* in A549 cells upon IL-1β stimulation, this result suggesting the role of NF-κB and AP-1 in regulating *Tgfb1* expression (75).

C-Jun protein is a component of the dimeric transcription factor activator protein 1 (AP-1) (76), it has been shown to play essential roles in most fibrotic conditions, which can activate multiple signaling pathways, resulting in the expression of many fibrogenesis-associated genes (77). C-Jun N-terminal kinase JNK1 has been reported to promote fibrosis in the lung (78), inhibition of c-Jun phosphorylation *in vivo* reduced fibrosis and mRNA levels of *Tgfb1* in the unilateral ischemia reperfusion injury model (79). C-Jun expression in non-parenchymal liver cells (NPLCs) promotes the fibrogenesis and was particularly correlated with liver fibrosis during non-alcoholic steatohepatitis (80). Moreover, studies have shown that c-Jun can be degraded by the ubiquitin-proteasome system as the substrate of Fbxw7 (81, 82), which was also confirmed by our study. Our data show that deletion of *Fbxw7* in macrophages does not affect the levels of P65, EGR-1 and their phosphorylation, but significantly increases the level of AP-1 transcription factor subunit c-Jun.

Notably, the published data from Wang et al. (83) showed that FBXW7 deficiency prevents fibrogenesis by increasing telomere capping, which is contrary to the conclusion of our finding. Several potential possibilities exist for this discrepancy. Wang et al. used a mouse model of Fbw7 conditional knockout in pulmonary alveolar epithelial type 2 (AEC2) stem cells, while we used a model of *Fbxw7* conditional knockout in myeloid cells. Those reverse effects of Fbxw7 in AEC2 stem cells and myeloid cells in regulating lung fibrosis indicated that the pathogenesis of IPF is complicated, it involves the abnormal repair of lung tissue injury by alveolar epithelial cells, the disordered differentiation and proliferation of fibroblasts, or the hyperactivated recruitment and activation of innate immune cells. The dysfunction in any cell type can lead to different outcomes of IPF. Moreover, subtle genetic background differences in the strains of the mice used and microbiome differences between the mouse colonies may also contribute to these discrepancies.

In conclusion, our study revealed that Fbxw7 plays a crucial role in regulating pulmonary fibrosis progression. Myeloid-specific deletion of *Fbxw7* exacerbates pulmonary fibrosis by inhibiting c-Jun degradation and upregulating TGF-β expression in macrophages.

## Data Availability Statement

The raw data supporting the conclusions of this article will be made available by the authors, without undue reservation.

## Ethics Statement

The animal study was reviewed and approved by Medical Experimental Animal Care Commission of Zhejiang University.

## Author Contributions

QW, JZ, and JH designed the research. JH, YD, GL, XS, WS, LL, and MX performed experiments and acquired and analyzed data. JZ, JH, and PX drafted the manuscript. QW revised the manuscript. All authors read and approved the submitted version.

## Funding

This work was supported by the National Natural Science Foundation of China (31870907, 81930041, 91842103), Natural Science Foundation of Zhejiang Province (Z19H100001).

## Conflict of Interest

The authors declare that the research was conducted in the absence of any commercial or financial relationships that could be construed as a potential conflict of interest.

The reviewer YS declared a shared affiliation, with no collaboration, with several of the authors, JH, YD, GL, PX, XS, LL, MX, QW, to the handling editor at the time of review.

## Publisher’s Note

All claims expressed in this article are solely those of the authors and do not necessarily represent those of their affiliated organizations, or those of the publisher, the editors and the reviewers. Any product that may be evaluated in this article, or claim that may be made by its manufacturer, is not guaranteed or endorsed by the publisher.
