# Supplementary material for: Myeloid Fbxw7 Prevents Pulmonary Fibrosis by Suppressing TGF-β Production
Source: Front Immunol. 2022 Jan 5;12:760138. doi: 10.3389/fimmu.2021.760138 (PMC8767095; doi:10.3389/fimmu.2021.760138)
Supplement: Supplementary file 1 [file DataSheet_1.pdf]

Supplementary Material

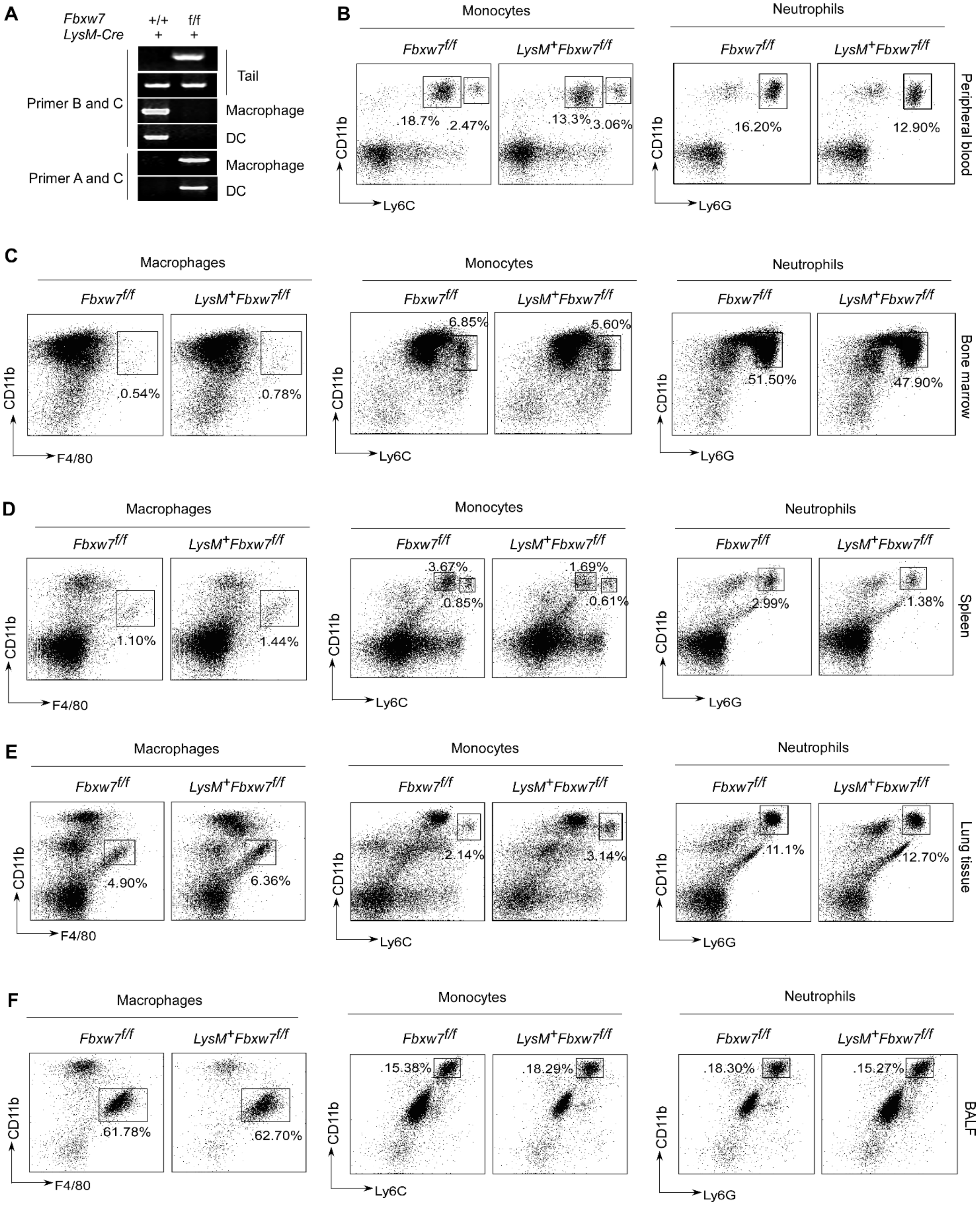

Supplemental figure 1. Myeloid-specific *Fbxw7* deletion does not affect the differentiation and development of myeloid immune cells in mice. PCR analysis of "floxed" and WT alleles (497 and 315 bp, respectively) using primer B and C, and determination of excision of exons 5 and 6 by primers A and C, which amplify 662 bp in the *LysM*$^{+}$*Fbxw7*$^{fl/fl}$ macrophages and DCs **(A)**. Flow cytometry analysis of CD11b^{+}Ly6C^{+} monocytes, CD11b^{+}Ly6G^{+} neutrophils and CD11b^{+}F4/80^{+} macrophages in peripheral blood **(B)**, bone marrow **(C)**, spleen **(D)**, lung tissue **(E)** and bronchoalveolar lavage fluid (BALF) **(F)** from

*Fbxw7^fl/fl^* and *LysM⁺Fbxw7^fl/fl^* mice. Data are the representative of three independent experiments.

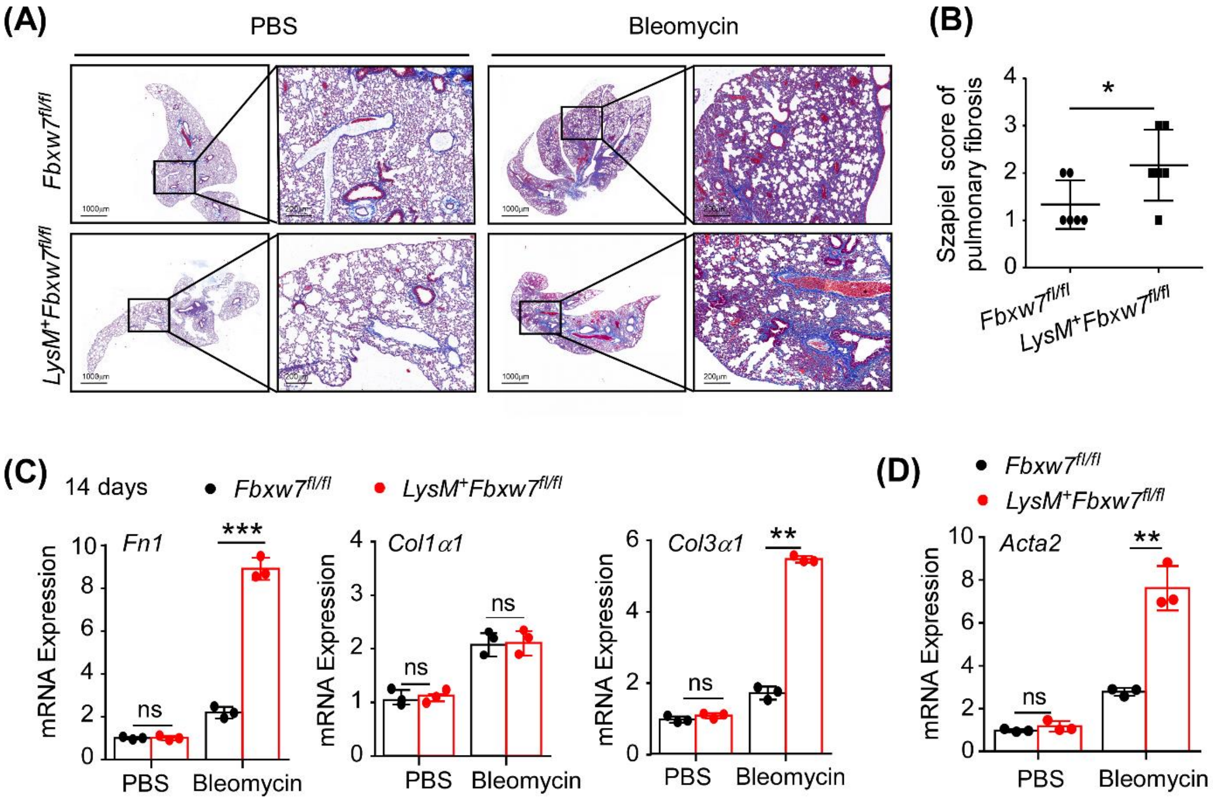

Supplemental figure 2. *LysM⁺Fbxw7^fl/fl^* mice show aggravated pulmonary fibrosis after a 14 d period of bleomycin-induced lung injury. *Fbxw7^fl/fl^* and *LysM⁺Fbxw7^fl/fl^* mice were administered bleomycin by direct endotracheal injection to induce pulmonary fibrosis. Three weeks later, the lung tissues were isolated for Masson staining and qRT-PCR analysis. **(A)** Masson staining of collagen fiber, scale bars 1000 μm (whole pulmonary section), and 200 μm (detail) and Szapiel score of pulmonary tissue **(B)** obtained from *Fbxw7^fl/fl^* and *LysM⁺Fbxw7^fl/fl^* mice. **(C)** The mRNA expression of collagen genes in lungs from *Fbxw7^fl/fl^* and *LysM⁺Fbxw7^fl/fl^* mice after bleomycin treatment for 14 days. **(D)** The mRNA expression of *α-SMA* (*Acta2*) in lungs from *Fbxw7^fl/fl^* and *LysM⁺Fbxw7^fl/fl^* mice after bleomycin treatment for 14 days. Data are expressed as mean ± SD of biological duplicates (n≥3) and are the representative of three independent experiments. P values were obtained using two-tailed Student's *t* test.*P <0.05,** P<0.01,***P<0.001.

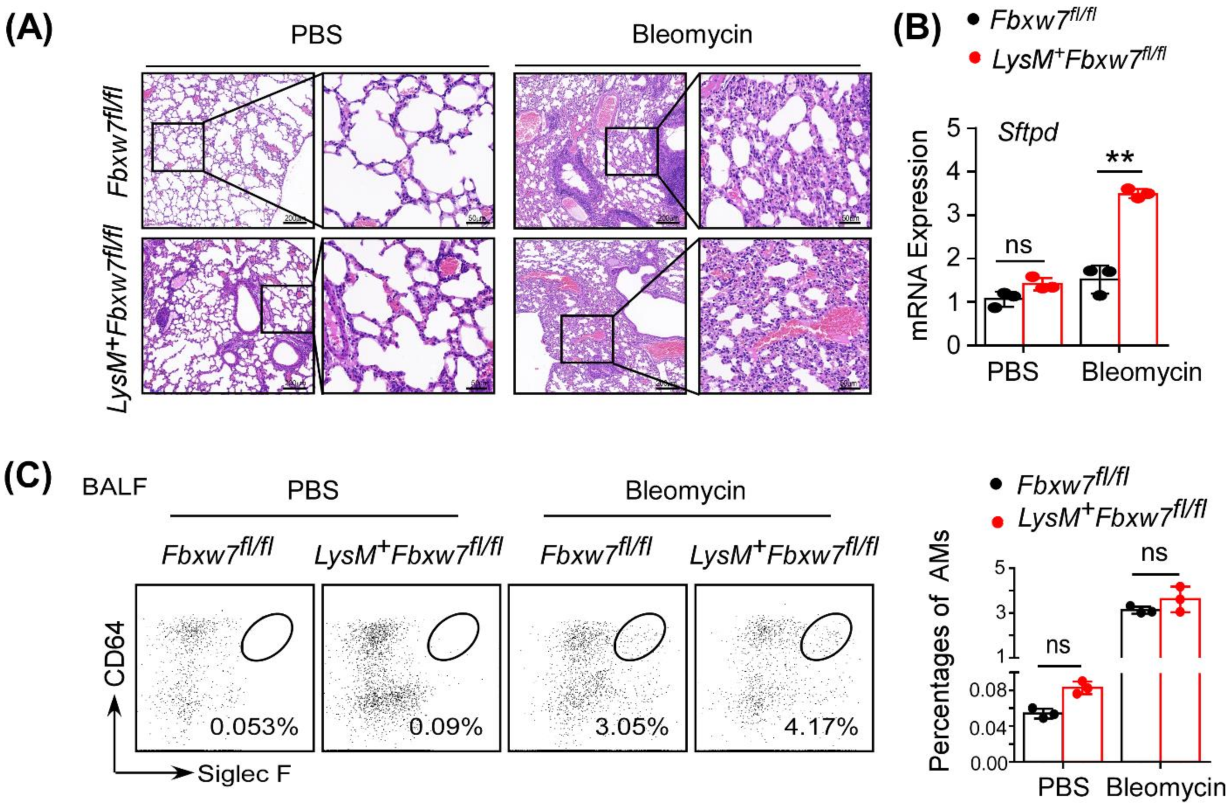

Supplemental figure 3. *LysM⁺Fbxw7$^{fl/fl}$* mice show more severe lung injury after a 14 d period of bleomycin treatment. (A) H&E staining of lung sections, scale bars 200 μm (whole pulmonary section), and 50 μm (detail). **(B)** qRT-PCR analysis of *Sftpd*. **(C)** Flow cytometry and statistical analysis of $CD64^{+}$Siglec F^{+} AMs in BALF. Data are expressed as mean ± SD of biological duplicates (n=3) are the representative of three independent experiments. P values were obtained using two-tailed Student's *t* test. ***P*<0.01.

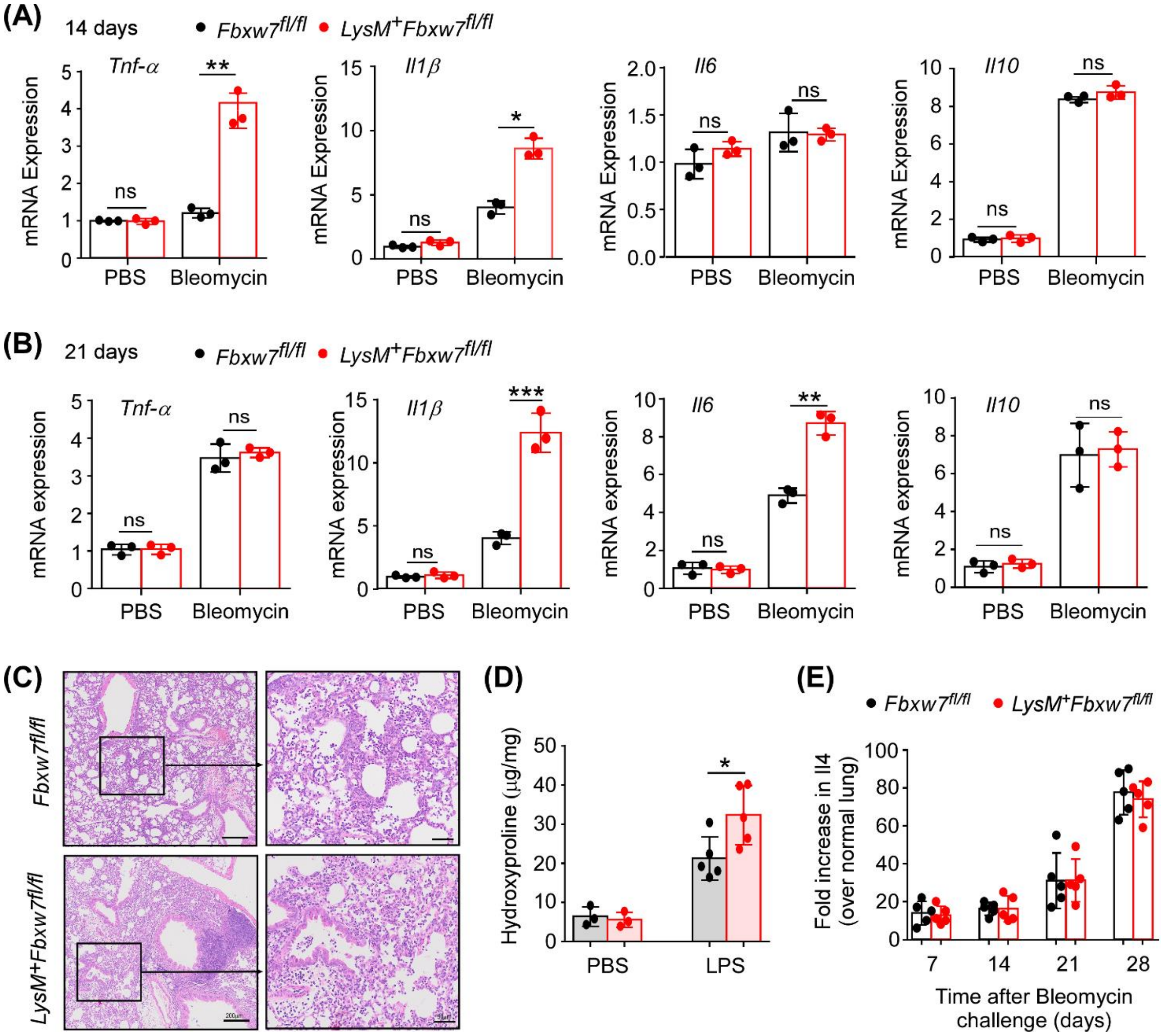

Supplemental figure 4. *LysM*$^{+}$*Fbxw7*$^{f/f}$ mice show more severe inflammation and lung fibrosis. *Tnf-α*, *Il1β*, *Il6* and *Il10* expression in lung tissue obtained from *Fbxw7*$^{fl/fl}$ and *LysM*$^{+}$*Fbxw7*$^{fl/fl}$ mice after a 14 days **(A)** and 21 days **(B)** period of bleomycin-induced lung injury. After LPS induced lung injury, lung tissue samples were harvested at 10 days. **(C)** Lung injury was assessed by H&E staining, scale bars 200 μm (whole pulmonary section), and 50 μm (detail). **(D)** Collagen deposition was assessed by Hydroxyproline content. **(E)** qRT-PCR analysis of whole lung samples for *Il4* mRNA expression on days 7, 14, 21, and 28 after bleomycin challenge. *Il4* mRNA expression in the whole lung samples was compared with mRNA expression in whole lung samples before the bleomycin challenge (day 0). Data are expressed as mean ± SD of biological duplicates (n≥3) are the representative of three independent experiments. P values were obtained using two-tailed Student's *t* test. *$P<0.05$,**$P<0.01$.

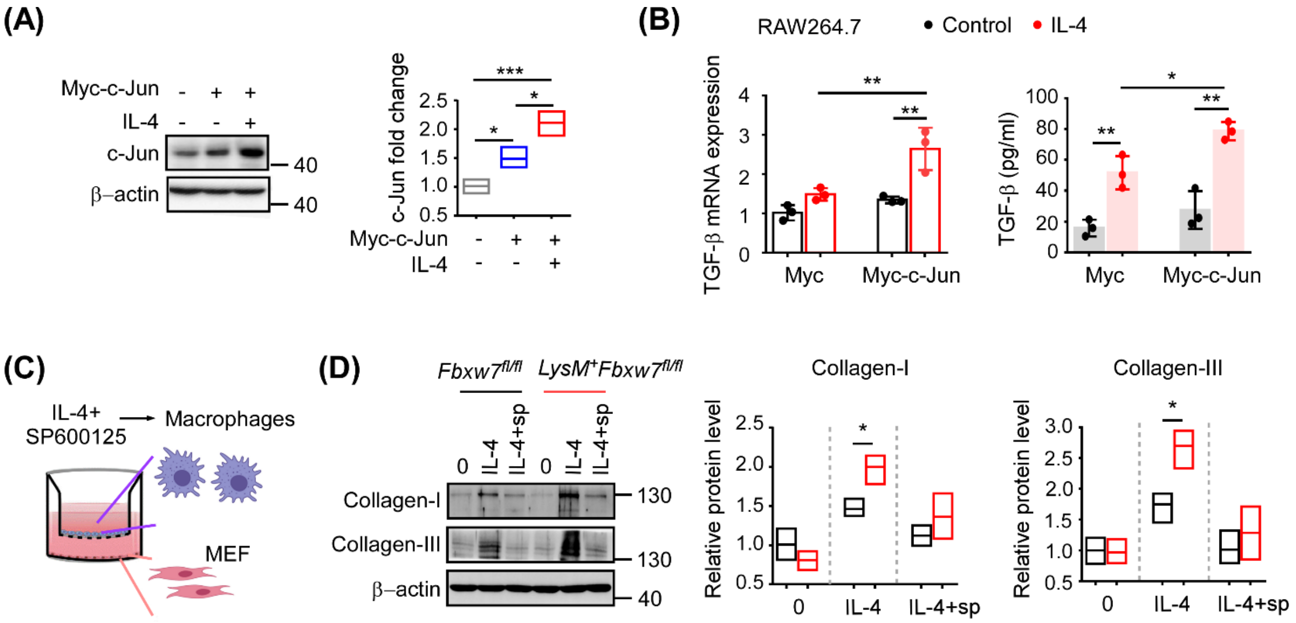

Supplemental figure 5. c-Jun in macrophages controls TGF-β expression. **(A)** Immunoblot analysis of c-Jun in lysates of RAW264.7 cells after transfection with 1 μg Myc-c-Jun and treated with IL-4 (30 ng/ml) for 0 or 8 hours. Densitometric fold change of c-Jun based on untreated group was calculated. **(B)** TGF-β expression in RAW246.7 cells was detected by qRT-PCR and ELISA. Data are expressed as mean ± SD of biological duplicates (n=3) are the representative of three independent experiments. Peritoneal macrophages from *Fbxw7^fl/fl^* and *LysM^+^Fbxw7^fl/fl^* mice were stimulated with IL-4 and treated with or without SP600125, then co-cultured with MEF cells for another 48 h. **(C)** The schematics of the approach. **(D)** Representative images of western blot of collagen-I and collagen-III were shown in co-cultured MEF cells. Densitometric fold change of c-Jun based on untreated *Fbxw7^fl/fl^* group was calculated. Densitometric analysis of target proteins was quantified by ImageJ, and the floating bars indicate the min, mean and max value of biological duplicates (n=3) are the representative of three independent experiments (A,D). P values were obtained using One-way ANOVA with Tukey's Multiple Comparison test (A,B) and two-tailed Student's *t* test (D). *P<0.05, **P<0.01, ***P<0.001.

Supplementary Table 1. Primers for qRT-PCR analysis

Gene	Forward primer (*5′-3′*)	Reverse primer (*5′-3′*)
Fbxw7	ACCAGCTCTCCTCTCCATTCT	CAACTTCTCTGGTCCGCTCC
Tgfb1	GAGCCCGAAGCGGACTACTA	CACTGCTTCCCGAATGTCTGA
Il1b	GCAACTGTTCCTGAACTCAACT	ATCTTTTGGGGTCCGTCAACT
Il6	CCAAGAGGTGAGTGCTTCCC	CTGTTGTTCAGACTCTCTCCCT
Il10	GCTCTTACTGACTGGCATGAG	CGCAGCTCTAGGAGCATGTG
Tnf-α	CCCTCACACTCAGATCATCTTCT	GCTACGACGTGGGCTACAG
Mmp9	CTGGACAGCCAGACACTAAAG	CTCGCGGCAAGTCTTCAGAG
Timp1	GCAACTCGGACCTGGTCATAA	CGGCCCGTGATGAGAAACT
Sftpd	AAGGTCCACGGGGTGAGAA	TTTGCCTTGAGGTCCTATGTTC
Acta2	CCCAACTGGGACCACATGG	TACATGCGGGGGACATTGAAG
Fn1	ATGTGGACCCCTCCTGATAGT	GCCCAGTGATTTCAGCAAAGG
Col3α1	GGCCTTTTACCTTTCACGGTG	TACGGCATTGTGGCTTCTCAA
Actb	AACAGTCCGCCTAGAAGCAC	CGTTGACATCCGTAAAGACC
